# Dignity in bodily care at the end of life in a nursing home: an ethnographic study

**DOI:** 10.1186/s12877-022-03244-8

**Published:** 2022-07-25

**Authors:** Bodil Holmberg, Tove Godskesen

**Affiliations:** 1 Department of Health Care Sciences, Palliative Research Centre, Marie Cederschiöld University, Box 11189, 100 61 Stockholm, Sweden; 2 Centre for Research Ethics & Bioethics, Department of Public Health and Caring Sciences, Uppsala University, BMC, Box 564, SE-751 22 Uppsala, Sweden

**Keywords:** End of life, Ethnography, Ethics, Dignity, Nursing home, Palliative care

## Abstract

**Background:**

Nursing homes (NHs) are populated by the frailest older people who have multiple physical or mental conditions and palliative care needs that may convey the violation of dignity. Although dignity is a commonly used concept and a core value of end-of-life care, it is assumed to be complex, ambiguous, and multivalent. Thus, the aim of this study was to explore aspects of dignity in older persons’ everyday lives in a NH.

**Design:**

A focused ethnographic study design.

**Methods:**

Data consisted of 170 h of fieldwork, including observations (*n* = 39) with residents (*n* = 19) and assistant nurses (*n* = 22) in a Swedish NH. Interviews were undertaken with residents several times (in total, *n* = 35, mean 70 min/resident). To study dignity and dignity-related concerns, we used the Chochinov model of dignity to direct the deductive analysis.

**Results:**

The study showed that residents suffered from illness-related concerns that inhibited their possibilities to live a dignified life at the NH. Their failing bodies were the most significant threat to their dignity, as loss of abilities was constantly progressing. Together with a fear of becoming more dependent, this caused feelings of agony, loneliness, and meaninglessness. The most dignity-conserving repertoire came from within themselves. Their self-knowledge had provided them with tools to distinguish what was still possible from what they just had to accept. Socially, the residents’ dignity depended on assistant nurses’ routines and behaviour. Their dignity was violated by long waiting times, lack of integrity in care, deteriorating routines, and also by distanced and sometimes harsh encounters with assistant nurses. Because the residents cherished autonomy and self-determination, while still needing much help, these circumstances placed them in a vulnerable situation.

**Conclusions:**

According to residents’ narratives, important dignity-conserving abilities came from within themselves. Dignity-conserving interventions did occur, such as emphatic listening and bodily care, performed in respect for residents’ preferences. However, no strategies for future crises or preparing for death were observed. To protect residents’ dignity, NHs must apply a palliative care approach to provide holistic care that comprises attention to personal, bodily, social, spiritual, and psychological needs to increase well-being and prevent suffering.

## Background

The older population of the world is growing quickly and will grow even more quickly in the future [1]. Because older people now tend to receive care at home for as long as possible, nursing homes (NHs) are populated by the frailest older people with multiple illness conditions, often with palliative care needs [1–3]. NH residents experience high symptom burden and psychological distress, which may cause a sense of hopelessness and of violated dignity [4]. Preserving the dignity of NH residents is the responsibility of healthcare staff and is a core value of the palliative care philosophy [5, 6].

Dignity is a central concept in many regulatory and ethics documents. The best-known of these is perhaps the 1948 Universal Declaration of Human Rights. The first article states that: “All human beings are born free and equal in dignity and rights” [7]. Later UNESCO declarations express similar principles, such as: “Everyone has a right to respect for their dignity and their rights regardless of their genetic characteristics”; further that “dignity makes it imperative not to reduce individuals to their genetic characteristics and to respect their uniqueness and diversity” ([8], Article 2). The declarations hold that all human beings are owed equal respect, irrespective of age, ethnicity, language, gender, sexuality, or abilities. In healthcare, dignity likewise plays an essential role in many policies [9–11]. Respect for a person’s dignity is thus central to NH care [12, 13]. Despite this, it has been claimed to be complex, ambiguous, and multivalent [14]. Some scholars have analysed dignity conceptually [15–20], while others have empirically studied how patients, healthcare professionals, or students use the concept [21–24]. The ambiguity has led some scholars to suggest that the concept has little ethical value, especially in healthcare, as the principle of respect for personal autonomy basically implies the same considerations [25]. In response, other scholars see dignity as having a “thicker usage”, more “closely tied to relational issues of upholding personal standards and avoiding humiliation”, and thus having “great relevance to medical ethics” ([26], p. 160).

Nordenfelt argues that respect for dignity is essential in all human interactions, not least in the care of older persons [15, 27]. Nordenfelt has distinguished four aspects of dignity: *human dignity*, *dignity of merit, dignity of moral stature*, and *dignity of identity*. *Human dignity* is described as being inherent to all human beings to the same extent and cannot be lost as long as a person exists. *Dignity of merit* is dependent on social rank and position and exists to varying degrees and over time. *Dignity of moral stature* results from moral deeds and can be reduced or lost through a person’s immoral deeds. *Dignity of identity* is tied to the integrity of the person’s body and mind and is dependent on each person’s self-perception. According to Nordenfelt, the *dignity of identity* aspect is the most significant in illness and ageing. Disability and constrained autonomy further affect a person’s identity and thus dignity. Therefore, *dignity of identity* is central to persons living in NHs because it can be shattered or lost by external events, disrespectful acts, illness, or old age. This kind of dignity can also vary over time and circumstances and is either promoted or taken away because of changes in body, mind, and situation [27]. NH staff must therefore act in ways that help to respect an older person’s sense of dignity and that align with a person-centred care approach [28].

Suffering from illness, disability, and old age may lead to a wide range of existential, spiritual, and psychosocial concerns, while experiencing dignity seems to mitigate suffering in end-of-life illness and death [29]. Nevertheless, respecting dignity might be a challenge because it is not explicitly clear what this requires in clinical practice.

Chochinov is one of few scholars who has developed an empirical model for the application of dignity in a palliative care setting, the so-called Dignity Model (DM) [30–32]. The DM aims to activate the principles of dignity in palliative care and bolster the person’s sense of dignity by inviting the individual to reflect on matters of personal importance while addressing sources of psychosocial and spiritual distress [30, 33]. The DM intends to develop a comprehensive set of measures evaluating dying persons’ quality of life and their required care regarding their physical, psychosocial, and spiritual sources of influence. The model describes dignity in three broad categories: ‘illness-related concerns’; ‘dignity conserving repertoire’; and ‘social dignity inventory’, and includes 23 perspectives. The DM also provides screening tools as guidance for healthcare staff to identify and provide suitable care regarding central physical, emotional, spiritual, or social concerns to avoid causing distress to dying persons and their families.

Upholding the dying person’s dignity is a core value in end-of-life care. Thus, clarifying this would inform healthcare providers and educators to attend to and respect the dignity-related needs of older persons with complex care needs. However, ethnographic research investigating the upholding of dignity in NH contexts is rare, and very little is known about using a dignity model to understand aspects of dignity from older persons’ perspectives in end-of-life care. Therefore, in this study, we studied residents in their daily NH setting by using an ethnographic method through participant observation and face-to-face interviewing to collect data.

### Aim

The aim of this study was to explore aspects of dignity in older persons’ everyday lives in a nursing home.

### Research questions

1. In what way do older persons’ narratives describe aspects of dignity?
2. What dignity-conserving interventions occur in end-of-life care observations?

## Methods

### Design

This ethnographic and deductive study was based on participant observations and interviews inspired by focused ethnographic principles [34, 35]. Focused ethnography has its roots in anthropology [35] and enables exploration of a particular issue in a specific setting. The method focuses on subcultural groups sharing particular traits instead of looking at whole societies [35, 36]. It is a recommended method in research involving older people [37] and palliative care [38] as it allows a deep understanding of a particular question and context and is carried out in an everyday setting. A deductive analysis approach was chosen as its structure was operationalized on the basis of an existing theory [39]. In this study, the theoretical dignity model (DM) by Chochinov was used [31].

### Setting

NH residents and assistant nurses were recruited from a public nursing home in a Swedish rural area. In Sweden, older persons’ care is governed by the Social Services Act and is mainly the responsibility of the municipalities, thus funded by taxes. The NH had four accommodation units, comprising 32 residents in total. Each ward shared a standard living room and kitchen. The residents lived in one-room flats, each with a private bathroom and kitchenette. The residential median length of stay was 17 months (range: 0.5–69, calculated from room-renting data from 2010 to 2017). Later data present a median length of stay of 24 months at Swedish NHs [40]. The NH was staffed by assistant nurses around the clock (Table 1). One registered nurse worked at the NH during the day, and one General Practitioner regularly performed a doctor’s round for three hours per week. Otherwise, both professionals were available on call.

**Table 1:** The distribution of assistant nurses during the 24-hour period

Working shifts	Time	Number of ANs/ shift
Daytime weekdays	7 am – 4 pm	2
Evenings weekdays and weekends	4 pm – 21 pm	1 + 1 ambulatory, covering all units
Weekends	7 am – 12 o’clock	2
Weekends	12 o’clock – 4 pm	1
Nights	9 pm – 07 am	2 covering all units

### Participants and data collection

Bodily care was defined as help with (un)dressing, mealtimes, transfer, and personal hygiene. The residents’ inclusion criteria were ≥ 80 years, ≥ 2 diseases, assisted in daily bodily care, permanently living in the NH, and able to verbally communicate their experiences. Twenty-two residents were asked to participate, one did not want to participate in interviews, and two were excluded due to rapid cognitive deterioration during the data collection period. Thus, nineteen residents participated in the study. Twenty-two assistant nurses participated in the observations and were observed in their interactions with residents. Assistant nurses had completed 2–3 years of formal high school education with a focus on care. In all, thirty-nine observations and thirty-five interviews (mean 70 min) were collected and audiotaped (Table 2).

**Table 2:** Participant characteristics of residents and assistant nurses at Swedish nursing home

	**Residents (*n*= 19)**	**Assistant nurses (***n***= 22)**
**Sex**		
Female	16	22
Male	3	-
**Place of birth**		
Sweden	18	17
Europe	1	4
Overseas	-	1
**Age**		
20–30	-	4
31–40	-	3
41–50	-	4
51–60	-	8
61–70	-	3
71–80	-	-
81–90	9	-
91–100	10	-
**Mean age/Standard Deviation **	90.5/85–98	47/26–63
**Degree of bodily help needs**		
Totally dependent	1	-
Help with shower	16	-
Manages to shower with support	3	-
Manages to use the toilet	7	-
Help using the toilet	12	-
Manages to brush teeth/face/shave	12	-
Help to dress/undress	14	-
Manages to dress/ undress	5	-
Help with food	10	-
Manages to eat unsupported	9	-
Help with medicines	19	-
Manages to walk with roller	7	-
Help to move in wheelchair	7	-
Manages to move independently in wheelchair	5	-
**Participation in observations**	17	22
**Participation in interviews**	15	-

Upholding respect for participants’ integrity and diminishing strength was crucial during data collection. Several residents were interviewed on several occasions. Therefore, some interviews were shorter and were performed in conjunction with observations, whereas most were longer and performed in private. An empirical question often used in a well-known dignity model is “What do I need to know about you as a person to take the best care of you that I can?” [41]. Because we focused on seeking insight into the older persons’ narratives of their experiences of receiving bodily care, the following questions were used: “What is it like to be a resident here?”, and “How does it feel to receive bodily care?” Follow-up questions were used when appropriate. The use of broad opening questions is typical in narrative data collection methods, as it prioritises the storyteller’s perspective [42]. The storyteller controls the direction, content, and pace of the interview, while the interviewer listens, avoiding interruptions. Thereby, the meanings that the storyteller assigns to the story might be elucidated in the narrative. The first author fully transcribed the interviews verbatim. The interview-data to be analysed comprised 356 pages (A4) of single-spaced text.

The observations followed assistant nurses as they interacted with the residents. The first author observed and interacted socially but did not participate in bodily care procedures [43]. A two-part protocol was used: the first described demographic data (location, time, and date), and the second described the observations in free text. The first author took notes, and fieldnotes were written immediately following the observations [35]. The study took place for six months in 2017, corresponding to 170 h of observations in bodily care situations, during both the day and at night. The transcribed observational data to be analysed comprised 68 pages (A4) of single-spaced text.

### Theoretical frame and data analysis

A deductive content analysis [39] was carried out, based on the model of dignity proposed by Chochinov [31]. The DM aims to improve patients’ sense of dignity, purpose, meaning, and self-worth and reduce psychosocial and spiritual distress. The model describes dignity in three broad categories: ‘illness-related concerns’; ‘dignity-conserving repertoire’; and ‘social dignity inventory’, guided by 23 perspectives (Table 3). The questions address those aspects of life that patients assess as being most meaningful in relation to their experience of dignity. To theorize the DM, we lean towards the descriptions of *human dignity*, as presented by Nordenfelt above [27]. As *human dignity* is an umbrella concept that is difficult to operationalize, we argue that issues concerning *dignity of identity* are processed through the DM’s perspectives 1–5, issues concerning *dignity of merit* are processed through the DM’s perspectives 3 A–C, while issues concerning *dignity of moral stature* are processed through the DM’s perspectives 3G and 5D.

**Table 3:** Chochinov model used for deductive analysis

A Model of Dignity and Dignity-Conserving Interventions for Patients Nearing Death			
	Factors	Dignity-Related Questions **(interviews)**	Therapeutic Interventions **(observations)**
Illness-related Concerns			
1. Symptom distress	A. Physical distress	How comfortable is the person?	Is there vigilance to symptom management? Is there a frequent assessment? Is there application of comfort care?
	B. Psychological distress	How is the person coping with what is happening to him/her?	Is a supportive stance assumed? Is there empathetic listnening? Is there referral to counseling?
	C. Medical uncertainty	Would the person like to know anything further about his/her illness? Does he/she have all the information that he/she feels is needed?	If requested – are accurate and understandable information and strategies to deal with possible future crises provided?
	D. Death anxiety	Would the person like to discuss things about the later stages of his/her illness?	
2. Level of independence	A. Independence	Has the illness made the person more dependent on others?	Is the person participating in descicion-making, regarding both medical and personal issues?
	B. Cognitive acuity	Does the person have any difficulty with his/her thinking?	Is delirium treated? When possible, are sedating medications avoided?
	C. Functional acuity	How much is the person able to do for him/ herself?	Are orthotics, physiotherapy and occupational therapy performed?
Dignity-conserving Repertoire			
3. Dignity-conserving perspectives	A. Continuity of self	Are there things about the person that disease does not affect?	Are those aspects of life that the person values the most acknowledged? Is the person seen as worthy of honor, respect and esteem?
	B. Role preservation	What were the most important things the person did before illness?	
	C. Of pride	What about self or life is the person most proud of?	
	D. Hopefulness	What is still possible?	Is the person encouraged and enabled to participate in meaningful or purposeful activities?
	E. Autonomy/control	How in control does the person feel?	Is the patient involved in treatment and care decisions?
	F. Generativity/legacy	How does the person want to be remembered?	Is the person active with life projects (making videotapes, writing letters, journaling)?
	G. Acceptance	How at peace is the person with what is happening to him/her?	Is the person supported in his/her outlook? Is the person encouraged to do things that enhance his/her sense of well-being (meditation, light exercise, listening to music, prayer) ?
	H. Resilience/fighting spirit	What part of the person is strongest right now?	
4. Dignity-conserving practices	A. Living in the moment	Are there things that take the person’s mind away from illness and offer comfort?	Is the person allowed to participate in normal routines or comforted in momentary distractions (daily outings, light exercise, listening to music)?
	B. Maintaining normalcy	Are there things the person still enjoys doing on a regular basis?	
	C. Finding spiritual comfort	Is there a religious or spiritual community that the person is or would like to be connected with?	Are referrals to chaplain or spiritual leaders made? Is the person enabled to participate in particular spiritual and/or culturally based practices?
Social Dignity Inventory			
5.	A. Privacy boundaries	What about privacy or body is important to the person?	Is permission asked to examine the person? Is draping done properly to safeguard and respect privacy?
	B. Social support	What people are most important to the person? Who is the person’s closest confidante?	Are there liberal policies about visitation?
	C. Care tenor	Is there anything in the way the person is treated that undermines his/her sense of dignity?	Is a stance where the person is treated as worthy of honour, esteem and respect adopted?
	D. Burden to others	Does the person worry about being a burden to others? If so, to whom and in what ways?	Are explicit discussions about these concerns with those they fear they are burdening encouraged?
	E. Aftermath concerns	What are the person’s biggest concerns for the people he/she will leave behind?	Are the setting of affairs, preparation of an advanced directive, making a will, funeral planning encouraged?

A categorisation matrix was developed in line with Chochinov’s DM [31]. The DM contains questions to be posed to a person. In our matrix, the original model’s questions and perspectives were slightly modified into questions, designed to be posed to the interview narratives and observational fieldnotes. For instance, the question “How comfortable are you?” was modified to “How comfortable is the person?” The model’s therapeutic intervention, i.e., “vigilance to symptom management”, was modified to “Is there vigilance to symptom management?”, as the observations corresponded to what takes place in bodily care.

The analysis started with BH thoroughly reading all interviews and observations to obtain a sense of the whole and to become familiar with the data. Answers to the matrix’s questions and screening tools were searched for in the data. Textual aspects replying to each unique question and its related screening tools were identified and placed in datasheets, one for each matrix question and its related screening tools. The categorization of data then followed the structure of the matrix, resulting in the three main categories and four sub-categories presented below. BH analysed the interview data and TG analysed the observations, in line with the structured matrix.

### Consideration of rigour

In establishing rigour in this study, the criteria for trustworthiness described by Lincoln and Guba were used [44]. Both researchers scrutinised each other’s perspectives and clarified interpretations throughout the analysis process. Triangulation is a method used for trustworthiness and to enhance the validity and rigour of the data analysis in qualitative research [45] as well as in ethnography [43]. Thus, to test the consistency of the findings, two sets of data were used. Further, stakeholder engagement served to triangulate the observations and interviews. Stakeholder engagement can increase transparency, and guide the research process [46]. Consequently, an external check on the preliminary findings and interpretations was performed, with help of a stakeholders’ group composed of one person > 80 years, one NH-assistant nurse, one NH-nurse, and one NH-manager, who each read the results and provided valuable insights on aspects that were important to elucidate in the study discussion. The manuscript was also reviewed by other researchers at a seminar. A clear description of the findings and detailed use of verbatim quotes supports transferability [39]. Dependability was reached by the transparent documentation of each step taken, from the start of the study to the reporting stages. Further, confirmability was established by the researchers’ mutual reflexion on all choices.

## Results

### Illness-related concerns

The illness-related concerns sub-category included problems that threatened or influenced residents’ experiences of dignity, separated into two parts: Symptom distress, and Level of independence.

#### Symptom distress: 1 A–D

The residents suffered from bodily distress, such as tiredness, pain, and loss of functions, isolating them from socialising with others.

I’ve lost my spark today, I couldn’t get up for lunch, because I was so tired that I just wanted to lie down and sleep. My body is exhausted, all the joints and muscles and … everything. Goodness me, what pain I am in. (Interview with resident, 89 years)

Aid equipment provided some comfort but did not always fit and sometimes hurt, but assistant nurses provided comfort care by using various types of lifting aid equipment and putting pillows in strategic positions.

The disease progression and loss of abilities caused boredom and dependence on others that psychologically threatened residents’ sense of dignity. For example, the loss of vision and hearing made them lonely and the unpredictable body caused a state of vulnerability. These problems could make life feel meaningless:

Life goes on, well sort of. I don’t get involved as much these days, I feel like I am done with life. Sometimes you are sad, but mostly you are quite … well, empty. It has just got to end. (Interview with resident, 87 years)

Some residents coped by paitently accepting their situation, feeling privileged to live in a safe environment. Others resigned to angst and rumination or coped by remaining attentive, trying to control assistant nurses’ work. Residents’ negative feelings were reinforced by a sense of inferiority and fear of annoying staff, especially when a lack of empathy was perceived. When residents mentioned feelings of agony, assistant nurses were observed to listen and pose probing questions:

Resident: I’m dying. Assistant nurse: No, who has said that? Resident: I have. Assistant nurse, leaning over the bed, asks: Do you feel unwell? Or just feel old? She speaks calmly and allows long pauses. Resident: I’m scared. Assistant nurse: I understand. You can only take it one day at a time, can’t you? (Observation of resident, 90 years)

Alternatively, they started to sing or make a joke. There was a lack of information provided to residents. They expressed ignorance about medication and carried with them questions regarding the origin of diseases and new symptoms.

I think I’ve been quite healthy actually, so why did I have a stroke now, I don’t understand? I took my Warfarin and had staff who gave it to me, so I didn’t miss any. I don’t understand what happened. (Interview with resident, 88 years)

Further, residents did not know why the physiotherapist did not show up for training or why the physician had seponated certain medication. Overall, they desired more health monitoring, i.e., blood pressure measurement, but perceieved it as difficult to make contact with the nurse. This made residents feel neglected, which threatened their dignity. The assistant nurses were not observed to perform any regular check-ups on pain or other health problems. The residents did not express feelings of agonizing about their approaching death, nor did they have a wish to talk about later stages of their illnesses. However, when interviewed, they openly shared their thoughts about nearing death. They talked about it as being at *the final station*. They expressed this as being sad, but still natural, as there is no future when being old. Being old was described as something unpleasant and hopeless that threatened their dignity. Some residents wished to die *on the spot* to reduce further suffering. Others avoided thinking or talking about dying, but expressed a fear of becoming more dependent. Some were hopeful and curious about dying, looking forward to reuniting with dead loved ones. The assistant nurses were not observed to provide any strategies for future crises, i.e., offering conversations about later stages of residents’ illness.

#### Level of independence: 2 A–C

The progressing illnesses threatened residents’ dignity by increasingly making them dependent on others for help with bodily care. They were also dependent on assistant nurses when interacting with others, as they were increasingly losing their ability to socialise. In care situations, the assistant nurses were observed to support residents’ decision-making by asking about their personal preferences. Conversely, they were also observed to negatively influence residents’ dignity by neglecting their desires concerning clothing or the timing of care interventions. No discussions concerning medical decisions were observed.

The experience of cognitive acuity varied among residents. Some claimed to have a clear mind, thus instructing assistant nurses and interacting with family, while others perceived themselves as increasingly inert and forgetful. They described difficulties in collecting their thoughts and understanding instructions. This made them feel stupid, wondering whether they were in the early stages of a dementia disease. However, no treatment of delirium was observed, other than the distribution of sleeping pills and medication to prevent anxiety.

Some residents had a high degree of functional acuity, with limited need for assistance in daily acitivites, while others were restricted to such self-management activities as combing their hair or brushing their teeth. The residents noticed when their skills deteriorated, and practised to get better, or found alternative ways to reach their goals.

I feed myself, even though I need to have a knife, fork and spoon, because if I have to use my left, I get all shaky. So, I cut the food first, then I take the spoon and push the food onto it. (Interview with resident, 88 years)

To help residents, assistant nurses were observed to call for physiotherapists when needed and they also performed regular exercise sessions at the ward.

### Dignity-conserving repertoire

The dignity-conserving repertoire included strategies to mitigate concerns that threatened or influenced patients’ experiences of dignity. This sub-category was separated into two parts: Dignity-conserving perspectives, and Dignity-conserving practices.

#### Dignity-conserving perspectives: 3 A–H

Residents described how they retained a variety of personal characteristics, seemingly unaffected by disease. While some struggled with shyness and low self-esteem, others described themselves as social and humorous. On the whole, they presented high degrees of self-awareness that seemingly helped them to define who they had been, and still were. Thus, disease did not affect their degree of religious beliefs, humour, psychological stability, self-esteem, ability to enjoy aesthetic aspects in life, or decision-making capability. As the foundation of this role preservation, they mentioned the impact of their childhood, their marriages, living in wealth or in poverty, and losses they had experienced.

*My son died in 1994. The doctor said that we will be able to sort it, but they didn’t. I can hardly explain, it was so terrible. A child is very hard to lose. He was in his 50s, but still …* (Interview with resident, 98 years).

They also mentioned important hobbies, such as dancing, cooking, and needleworking. Some described life’s setbacks as natural challenges, while others were disappointed and felt unfortunate. However, they were proud of having coped independently and with endurance. Further, they were proud of their children, their careers, and talents for which others had credited them. At the NH, they were proud of still being active, i.e., by solving cross-words, knitting, or nursing plants. They also took pride in being able to teach assistant nurses how to perform complicated medical measures.

They come in, so I get to empty (the stoma). They haven’t seen this sort that I have, so I have to tell everyone about it. Him last night said that I told him exactly how he should do it (smiles and chuckles). They have to learn what to do. (Observation of resident, 91 years)

They also enjoyed seeing objects they had crafted with their hands. The observations showed that assistant nurses respected residents by listening when they signalled their need to talk, by asking for residents’ preferences on care, and by doing appreciated things, such as nail care. But they were also observed to neglect highly valued aspects of residents’ lives, e.g., when denying them help with their plants.

 While living in a nursing home, residents described that it was still possible to be stimulated by socialising with others, and participating in arranged activities. Further, by doing needlework, listening to the radio and watching TV, and performing physiotherapy in a hope to regain abilities. It was still possible to decide what clothes to wear, when to have a haircut, and independently perform certain parts of the bodily care. Some looked forward to the rare occasions when assistant nurses took them out for a walk or sat down to talk.

What is nice sometimes is when the staff sit with us. In the evenings it can be that some of them have a sandwich and a coffee when I sit (alone) out there. That is nice and enjoyable, so we can talk. (Interview with resident, 89 years)

In the observations, assistant nurses were seen to bring residents to activities such as group exercise sessions, dance events, and bingo sessions.

Residents with low functional capability described having no control. They were limited, supervised, and locked in, treated like a package without any impact on life. All residents experienced functional limitations, which made them feel unsuccessful and humiliated by their own bodies.

The assistant nurse helped me with a bra I was annoyed with. It bothers me that I can’t do it myself. I can’t control my body. I feel upset and annoyed that I can’t control necessary things. It feels degrading. (Interview with resident, 87 years)

To be autonomous, some residents steered assistant nurses and got their own way through humour and by adopting a good attitude. A wheelchair meant self-determination to some, as it allowed them to come and go as they wished. One way to gain control of their existence was by adapting to the ward’s routines, i.e., visiting the toilet when knowing that assistant nurses had time. They described their own room as a place of self-determination and relaxation. However, in the observations, residents were not seen to be involved in discussions concerning treatment, and they were not always asked about their preferences on care.

None of the residents mentioned how they wanted to be remembered, and they were not observed being active with life projects, i.e., journaling.

Being old and unable to control the body, residents claimed it necessary to become at peace with their help-needs and make the best of the present. They were at peace with using transfer equipment, as they did not want assistant nurses to get hurt helping them. They accepted plain hairstyles as assistant nurses did not have time to help them. They wore comfortable, pyjama-like clothes, as that made assistant nurses’ work easier. Living in a NH meant being safe, looked after by kind and polite assistant nurses, having plenty of time to spend on oneself. Thus, being this old and at the end of life, existence could not be better. The days were dull and residents missed having someone to talk to, but at least they had company. The strongest characteristics of the residents were their inner qualities. They had feelings of being good enough for others to accept. They had a strong urge to manage independently and sought clever solutions to everyday challenges in order to remain as independent as possible.

It happens sometimes that I drop the grabbing tongs, but then I do this (glides forward on the wheelchair seat, then sits on the edge and bends forward to reach the floor). I have done this many times. If I am lucky, I can reach it then. (Observation of resident, 85 years)

The observations did not convey encouragement regarding residents’ personal interests. Instead, activities arranged at the ward were offered. Assistant nurses were seen to arrange appointments at the hairdresser when residents asked for it, but on a daily basis they arranged hairstyles and chose clothes for residents without asking.

#### Dignity-conserving practices: 4 A–C

Social events, such as exercise sessions, outdoor walks, and visitors, took residents’ minds away from illness, offered comfort, and strengthened their dignity. Having coffee with others, joking, and engaging in deep conversations worked in the same way.

On Sunday, I had an old colleague here with me. It was so incredibly enjoyable! At night I lay down and thought of lots of things, because that’s how memories come to life. (Interview with resident, 88 years)

Tasty food, home-baked cookies, and occasionally a little glass of whisky, brightened up life. A visit to the hairdresser was appreciated as a moment of personal attention. Another way of getting away from illness and boredom was to sleep as much as possible. On a more regular basis, residents enjoyed their weekly shower. Getting up in the morning and being able to watch other people were other daily amusements. Daily activities, such as reading papers, knitting, and day-dreaming were appreciated. The observations showed common meal and coffee times, but also residents who sat alone, distanced from ongoing conversations. There was nothing such as daily outings.

No residents expressed a desire to be connected to religious or spiritual communities. Few viewed themselves as religious, but, conversely, they did not deny the existence of a God. Nevertheless, if life would get too difficult, they would pray to God to let them die. The observations did not show any spiritual practices, but assistant nurses were seen to practically help residents to vote in a church election. Cultural practices such as musical entertainment were offered.

### Social Dignity Inventory

#### 5 A–E

The residents had been raised to cover private body parts. This had made it important to avoid nakedness before a person of the opposite sex.

I don’t want any of the men to see me naked. Absolutely not! It feels very uncomfortable. (Interview with resident, 98 years)

Residents were taught to take care of their personal hygiene independently, which generated their efforts to participate in their bodily care. Therefore, failing to use the toilet was described as the most humiliating thing that could happen. To ease embarrassment, they desired to be informed before care actions took place. No observations showed assistant nurses asking for permission before examination or assistance, but they did inform residents while performing them. Respect for integrity was lacking, as toilet doors were left wide open when residents sat naked on the toilet or in the shower.

Residents’ children were described as being their closest confidantes, who provided valued company and comfort. They were trusted to run residents’ finances and buy their clothes and make the residents’ rooms homier. In the observations, residents’ children were seen at various times, as visitation policies were liberal.

The residents were often left waiting for help, which could mean that they wet themselves. Long waiting periods made them feel like children, and having their bottoms wiped by someone of the opposite sex evoked feelings of being disgusting. Dignity was undermined when assistant nurses denied desired help, and when they asked residents to try it themselves first, as if they had not already. Understaffing led to inhibited practice and few outdoor-walks, and some residents could not remember the last time they had been outdoors. Insufficient staffing also restricted residents’ opportunity to shower once a week, which was considered too little. For practical reasons, residents sat on the toilet seat while showering. This was humiliating, as they did not enjoy washing in the same place where they empty their bowels or bladder. Further, the strict shower routines made showering feel impersonal.

I feel like some kind of cattle. In some way it feels a bit impersonal, when you just like come along like sheep and just stand there and let them keep on rubbing in soap and showering you. (Interview with resident, 87 years)

Clothes were not washed at the desired rate, thus residents felt filthy. Assistant nurses were always in a hurry, which had caused fall injuries and fear of falling. Further, it made residents with extensive-care needs feel neglected, while residents with less needs felt abandoned and lonely. The way work was organised could make residents feel devalued.

*Yesterday when I was brushing my teeth, I was made to sit on the toilet. I thought – now this has gone too far! To sit and spit into a little bowl, that’s awful. It feels like I am about as much worth as a … as a pig!* (Interview with resident, 89 years)

However, residents did not complain, as that rendered sharp admonishments from assistant nurses. The assistant nurses’ stance was described as being mentally distanced and exhortative, i.e., when telling residents to go to bed or reprimanding them for overusing the call button. This made residents feel supervised and denied their empowerment. Residents suffered when hearing assistant nurses’ mutual complaints about their body weight, and futher humiliated them by wearing gloves when washing their faces. When assistant nurses were in a bad mood, residents noticed a poor atmosphere and reckless care, which made them feel devalued. Likewise, they felt devalued when assistant nurses disbelieved them of being in pain, or passed on information that the residents had entrusted them in confidence. The observations presented situations where assistant nurses respected residents’ desire to sleep a bit longer, and hugged them or performed other things they had asked for. They also revealed situations when assistant nurses talked over residents’ heads, neglected what they said, neglected to perform mouthcare, omitted to wipe after micturation, or left residents in the middle of care without telling them why or when they would come back.

Residents feared being a burden to others, as that felt like being cruel. They felt guilt towards their children, who had already helped them so much. They excused their children for not visiting as often, as they had their own lives to live and families to care for. Residents also hesitated to disturb assistant nurses, because they were so busy. When having to ask for help, they felt like a nuisance. They tried to restrict their demands, especially in mornings and evenings, when all residents needed help simultaneously. Thus, they accepted eating cold food and tried to regulate their bodily needs.

You adapt to the toilet visits, otherwise you have to get help all the time. I think it’s enough if I get help to the toilet 4 times a day. That’s enough. I do that for everyone who works here, people like it if we cooperate and I just go with the flow. You don’t want to disturb people all the time when they are running around like crazy. (Interview with resident, 91 years)

Residents also tried to ease assistant nurses’ working situation by being jovial to help them thrive at work. The observations showed their desire to unburden assistant nurses by being willing to wait if necessary. No observations showed assistant nurses initiating or encouraging conversations about residents’ fear of being a burden.

Except for desiring the best for their children, no residents expressed any concerns about their passing. They were not observed to plan for their death, nor were assistant nurses observed to encourage such planning.

## Discussion

The results showed that residents suffered from *illness-related concerns* that inhibited their possibilities to live a dignified and social life at the NH. The results pinpoint residents’ failing bodies as the most significant threat to their dignity. Their ongoing loss of abilities and fear of becoming more dependent caused agony, loneliness, and meaninglessness. This is congruent with earlier research, describing loss of autonomy as being a worry to residents [47], because persons living in the interaction between suffering and health strive to find dignity [48]. More precisely, this exemplifies residents’ loss of dignity of identity, as disease and disability threaten to demolish their self-respect and humiliate them [27]. Different personalities coped differently, but they did not receive the needed support from assistant nurses, who were described as always being in a hurry. Empathetic listening occurred when residents needed help to cope, but they were also met with a lack of empathy or jokes. Humour can be used to balance inequalities and create connections between people, thereby playing down feelings of vulnerability by pointing at the absurdity of a situation [49], however, only provided it is used when knowing how the resident perceives the situation. Humour can also be used by assistant nurses as a means to avoid involvement in existential conversations [50]. Further, it may indicate an ageistic approach, where assistant nurses use humour as a socially acceptable form of discrimination to circumvent residents’ requests when believing themselves to know what is the best practice [51]. As the results demonstrated, residents felt inferior to assistant nurses, further doubting their cognition, thus they were not likely to object if humour was used in a derogatory way. In congruence with this, earlier research shows that residents try to maintain a façade of normality and avoid complaining to preserve their dignity [52]. This may explain why residents in this study avoided asking anyone about diseases and treatments. It may also explain why residents did not talk about fears of being a burden to others.

Although residents’ bodies were the largest violators of dignity, the most *dignity-conserving repertoire* came from within. It was apparent that they had different personalities, coloured by their previous lives. Despite this, they were proud of having coped independently and with endurance. Their acquired perspectives of self-knowledge provided them with tools to distinguish what was still possible from what they just had to accept. Thus, even those with extensive care needs and no control found ways to escape negative thoughts and find meaning in everyday life. This ability may result from inner creativity, helping residents to remain self-dependent, even if their bodies fail [53]. Residents in this study appeared to have found dignity by accepting disease and deterioration with a fighting spirit, which aligns with research with younger people with multiple sclerosis [54].

Further, residents in this study were proud of earlier achievements as well as current abilities. This is positive, as the dwelling in pleasant memories, the uphold of interests, and the maintenance of curiosity regarding preservation of zest for life based on individual prerequisites can confirm residents’ dignity [55]. All residents were retirees, thus without the dignity of merit connected to a position in society. However, we suggest that, in facing their daily challenges with patience and insight, stemming from lifelong experiences, the older persons possess a specific *dignity of merits*. This type of dignity, according to Nordenfelt, assumes the wisdom of life that only a person who has lived a long life can achieve [27]. This wisdom generates knowledge of human life in general, and helps older persons solve practical problems because they have solved similar problems before. Although their intelligence and experiences may vary, they all have this type of dignity by virtue of high age. This makes them worthy of dignified encounters. Adding to their specific dignity of merits, younger people need to offer this group of older people specific considerations for what they have achieved in life, for instance, as citizens and parents. As residents inevitably approach their deaths, they are undoubtedly in a vulnerable situation and thus in need of special regard. However, residents’ work to uphold inner dignity-conserving perspectives was rarely supported by assistant nurses, who were busy performing practical tasks. This underlines the notion that residents and assistant nurses in this study did not share the same reality, although coexisting under the same roof. This may be explained as organisational demands being in conflict with personalised care based on residents’ personal needs and preferences [56]. The results of this study show that the only dignity-conserving practices that assistant nurses provided were those belonging to the ward’s routines. This may threaten residents’ dignity, as not maintating a sense of personal self can result in feelings of being unseen and erased as a person [57]. Therefore, the activities offered at nursing homes need to become more individualised.

Socially, residents’ dignity depended on assistant nurses’ routines and behaviour. As the results show, their dignity was violated by long waiting times, lack of integrity in care, and deteriorating routines, and further by distanced and sometimes harsh encounters with assistant nurses. As residents cherished autonomy and self-determination while still needing much help, these circumstances placed them in a vulnerable situation. Having dignified treatment is vital, as freedom from pain, having clean clothes, treatment in respect of privacy, and human contact is central to the experience of dignity [57]. Further, the attitudes of assistant nurses are vital for dignifying care, as empathic encounters and respectful manners enhance dignity [58]. Here, this study provides a long line of examples demonstrating when residents’ dignity of identity was violated by external events [27], i.e., when assistant nurses treated and met them with lack of respect. When discussing undignifying experiences, several residents used metaphors [59]. This may be interpreted as part of a coping process, as humorous personifications can provide strategies to reduce the psychological impact of negative experiences, maintaining a sense of self and control [60]. Residents’ exposure to care grew more significant due to their efforts to avoid being a burden and their self-imposed task to behave jovially with assistant nurses by taking their needs into account before their own. This can be described to manifest residents’ *dignity as moral stature* [27]. Further, this might have helped residents to keep their self-respect and sense of dignity. Alternatively, not protesting when their dignity was violated may be explained by fear of being punished by the withdrawal of bodily care in vulnerable situations [61]. It was also evident that residents felt supervised, in need of more help to socialise and daily outings. Thus, they needed more attention to thrive and avoid feelings of abandonment and loneliness. Such a lack of freedom and recognition of residents as individual autonomous people can make them feel entrapped in a prison without bars, which can severely threaten their dignity [62]. The fact that residents did not appear to plan for their death, or worry about those they would leave behind, may be due to their energy-demanding struggles to safeguard dignity in everyday life. As residents at the end of life need to turn inwards and reflect on life to reach peace in the midst of health complaints and functional limitations, there is also a need to involve the concept of palliative care when providing care to very old people [52]. This implies the provision of holistic care that includes attention to bodily, social, spiritual and psychological needs to increase well-being and prevent suffering [63]. In the end, this would mean respecting residents’ *human dignity*, as this type of dignity is universal to all humans, independent of merits, inner thoughts, or others’ behaviour [27]. Thus, by focusing on therapeutic interventions regarding residents’ *dignity of indentity, identity of merit* and *indentiy of moral stature*, the vague concept of *human dignity* can be operationalized.

### Limitations

This study had some methodological limitations. First, most participants were women. This may have resulted in a female perspective of dignity-conserving interventions. A male perspective might have added other aspects. Alternatively, women generally live longer than men [64], and, as NHs foremost are populated by women, a female perspective is important. Second, social desirability bias must always be considered in studies [65]. This may have influenced residents to respond in a way they believed more appropriate or socially acceptable, resulting in the withholding of true thoughts and feelings. To reduce this, the first author spent a considerable amount of time in the NH, in fact, 170 h over six months, which provided a rich amount of data and a great depth of understanding of the aspects that influence the residents’ dignity. Third, data collection took place in 2017. This might appear to be a limitation; nevertheless, the conditions concerning staff and median length of stay in NHs in Sweden are similar today. Further, the insights revealed here add valuable knowledge to inform how dignity is embodied in NH practice.

### Recommendations for practice

To protect residents’ dignity, assistant nurses need to be aware of residents’ vulnerability, unique personalities, and preferences. Consequently, we suggest that assistant nurses be given opportunities for meaningful conversations and interactions with residents. Such initiatives have the prospect of helping assistant nurses to develop greater awareness of individually adapted dignity interventions. Despite the results of this study, much work remains to further develop dignity care. This could be done by using DM in randomized clinical trials in nursing home practices on a larger scale to test and develop robust evidence-based and dignified palliative care.

## Conclusions

According to residents’ narratives, important dignity-conserving abilities came from within themselves. Dignity-conserving interventions such as emphatic listening and bodily care, performed in respect of residents’ preferences, occurred. However, no strategies for future crises or preparing for death were observed. To protect residents’ dignity, NHs need to apply a palliative care approach to provide holistic care that comprises attention to personal, bodily, social, spiritual, and psychological needs to increase well-being and prevent suffering.

## Data Availability

The Swedish datasets analysed in the current study are available upon reasonable request from the first author.
